# The Bouba-Kiki Phenomenon Tested via Schematic Drawings of Facial Expressions: Further Validation of the Internal Simulation Hypothesis

**DOI:** 10.1177/2041669516631877

**Published:** 2016-02-29

**Authors:** Sethu Karthikeyan, Bianca Rammairone, Vijayachandra Ramachandra

**Affiliations:** Communication Sciences and Disorders Program, Pace University, NY, USA; Department of Communication Sciences and Disorders, Marywood University, Scranton, PA, USA

**Keywords:** bouba-kiki, sound-shape mapping, cross-modal activation, mirror neurons

## Abstract

Sound-shape associations involving consistent matching of nonsense words such as ‘bouba’ and ‘kiki’ with curved and angular shapes, respectively, have been replicated in several studies. The purpose of the current study was to examine the robustness of previously noted sound-shape associations when shape variations (angular and curvy) are embedded in schematic expressions of emotions (sad and happy). Results revealed consistent matching tendencies based on sound-emotion expression mapping irrespective of the particular shape of the expressions. We suggest that internally simulating the facial expressions/oral gestures may have played a significant role in driving the matching preferences.

Recent studies that reveal consistency in different kinds of sound-meaning associations, and the increasing scope of multimodal activations in the brain raise questions about the extent of arbitrariness in language (e.g., see review in Schmidtke, Conrad, & Jacobs, 2014). When angular and rounded shapes are presented with nonsense words such as bouba and kiki, 95% of adults match the rounded shape with bouba and the jagged shape with kiki—the bouba-kiki phenomenon (Ramachandran & Hubbard, 2001; for original work, see Köhler, 1929), an effect that has been replicated cross-linguistically (e.g., Bremner et al., 2012). As well, toddlers and infants display similar matching tendencies to those of adults (Maurer, Pathman, & Mondloch, 2006; Ozturk, Krehm, & Vouloumanos, 2012) suggesting that the matching biases may be innate and not learned via experience. Whereas a few studies have found consonant-driven matching patterns (e.g., Nielsen & Rendall, 2013), Spector and Maurer (2013) found that even when the consonant environment was kept constant, toddlers demonstrated consistent vowel-shape matches of /i/ (as in beet) and /o/ (as in boat) with angular and curvy images, respectively.

Consider also that the articulatory gestures for /i/ and /o/ are similar to the lip movements in a smile and a frown (vocal tract shortening and lengthening) leading to comparable acoustic characteristics—raising and lowering of filtered frequency components referred to as formants (e.g., Raphael, Borden, & Harris, 2011). Listeners are able to accurately identify speech samples spoken with a smile and those spoken with a frown (Tartter & Braun, 1994). Additionally, the vowels /i/ and /o/ have been associated with pleasantness and gloominess, respectively (e.g., Newman, 1933). These findings point to the possibility that nonsense words with vowel sounds /i/ and /o/, as those mentioned earlier, may be non-arbitrarily linked to smile and frown expressions. The purpose of the current study was to examine the robustness of previously noted sound-shape associations when shape variations were embedded in schematic expressions of emotions (see Figure 1).

**Figure 1.**
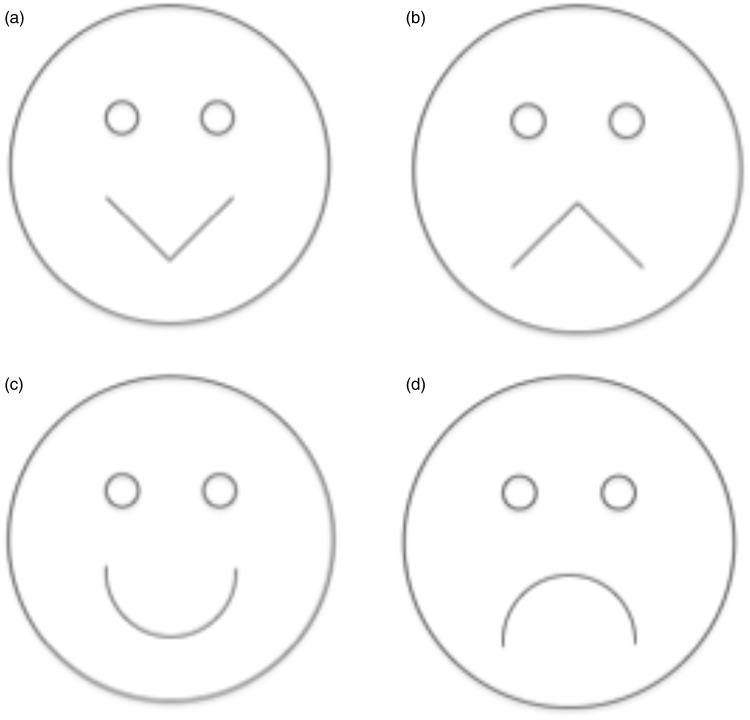
Schematic drawings presented via SuperLab 5; (a) happy angular, (b) sad angular, (c) happy curve, and (d) sad curve.

Ramachandran and Hubbard (2001) proposed a mirror neuron-based cross-modal activation hypothesis to explain the bouba-kiki effect; the internal simulation of the appropriate articulatory gesture of the auditory stimulus is mapped onto specific phonemic inflections, which then are non-arbitrarily linked with specific shapes or images. In the current study, while the facial expression of a smile or a frown may be internally simulated in addition to the motor patterns of the aurally presented words, the exact shape of the expressions may not be amenable to simulation because angularity variations such as the ones examined in the study are not naturally occurring or socially relevant (e.g., Oberman & Ramachandran, 2007). The resulting matching tendencies, therefore, must reflect consistencies between the corresponding acoustic characteristics of the emotional expression and of the auditory stimulus. For example, a sad angular/curvy face matched with words containing the /o/ vowel sound. If, on the other hand, simulations of facial expressions of emotions do not occur, the variations of the facial expressions in the angularity curviness dimension must influence the matching preferences in a similar manner to the established matching tendencies between sounds and random angular-curvy shapes in earlier studies. For example, a sad/happy angular face matched with words containing the /i/ vowel sound.

Each of the four computerized schematic drawings of faces was presented individually three or four times with randomly chosen word pairs (see Table 1). The aural presentation of the word pairs was counterbalanced across the four faces for the order of /i/ and /o/ vowel sounds and for the consonant environment. The institutional review board approved the study; 50 participants between 18 to 24 years (45 females and 5 males) were asked to match the happy/sad face with one of the words in the presented word pair by clicking on the appropriate text options: WORD 1 or WORD 2.

**Table 1.** Word Pairs Created Using Cepstral David (Swifttalker) Separated by 500 Milliseconds.

Consonant environment 1	Consonant environment 2
bibi-bobo	fifi-fofo
bobo-bibi	fofo-fifi
didi-dodo	kiki-koko
dodo-didi	koko-kiki
lili-lolo	titi-toto
lolo-lili	toto-titi
mimi-momo	zizi-zozo
momo-mimi	zozo-zizi

As Figure 2 shows, the happy angular face and the happy curve face was each matched more frequently with words containing the /i/ vowel sound than with words containing the /o/ vowel sound. The preference for the /i/ vowel sound did not differ between these two faces (paired *t* test, *t*_(49)_ = 1.59, *p* > .05). The sad curve face and the sad angular face was each matched more frequently with words containing the /o/ vowel sound. The preference for the /o/ vowel sound did not differ between these two faces (paired *t* test, *t*_(49)_ = 0.35, *p* > .05).

**Figure 2.**
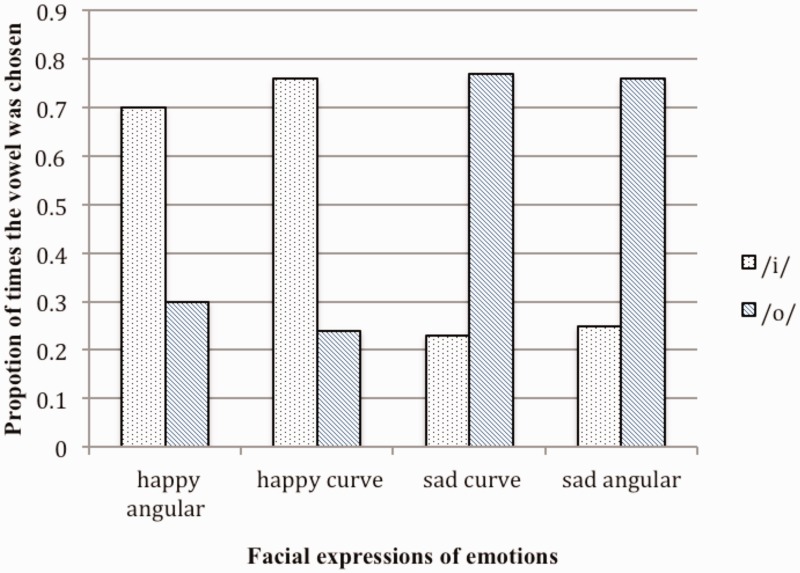
Proportion of times the vowels /i/ and /o/ were chosen for each of the faces.

If the acoustic characteristics associated with the exact shape of the facial expressions were being mapped onto the acoustic characteristics of the words presented aurally, the shape differences within each emotional category (curved vs. angular versions of happy and sad faces) would have resulted in a notable difference in the choices of vowel sounds; however, a difference did not emerge. We cannot rule out the potential influence of emotional contagion (e.g., Lundqvist & Dimberg, 1995), in that the happy “feeling” on seeing a happy face may have been mapped onto the “pleasant” sounding /i/ and the sad feeling on seeing a sad face was matched with the gloomy sounding /o/ (e.g., Newman, 1933).

In sum, the current study in conjunction with the previous ones on sound-symbolism demonstrate that cross-modal matching may allow for non-arbitrary associations of the vocal–verbal signal with aspects of inanimate and animate entities (including the self and others). When two or more aspects co-occur, these may compete with one another or one may supersede the others in guiding the associations; in the current study, the facial expressions took precedence over the angularity or curviness of the expressions. Also, there exists the possibility of covert imitation of the oral gestures alone (retraction of lips and rounding or protrusion of lips), devoid of any emotional meaning, to aid this kind of auditory visual matching task (see Studdert-Kennedy, 2000, 2002, for a discussion on the evolution of vocal imitation as a step toward promoting arbitrary linkages between signals and messages or referents). Considering the larger scheme of things, it is reasonable to deduce that a communication system that is based heavily on non-arbitrariness or iconicity could lead to ambiguity (e.g., Pinker & Bloom, 1990), and limited expressive scope (e.g., Studdert-Kennedy, 2000, 2002), a few possible factors that may have favored arbitrariness in the evolution of the human capacity for language.
